# Discrimination and Racial Inequities in Self-reported Mental Health Among Immigrants and Canadian-Born Individuals in a Large, Nationally Representative Canadian Survey

**DOI:** 10.1007/s40615-024-02128-4

**Published:** 2024-08-20

**Authors:** Kathleen S. Kenny, Susitha Wanigaratne, Lisa Merry, Arjumand Siddiqi, Marcelo L. Urquia

**Affiliations:** 1 Manitoba Centre for Health Policy, Department of Community Health Sciences, Max Rady College of Medicine, Rady Faculty of Health Sciences, University of Manitoba, Winnipeg, Canada; 2 Edwin S.H. Leong Centre for Healthy Children, SickKids Research Institute & the University of Toronto, Toronto, Canada; 3 ICES, Toronto, Canada; 4 Faculty of Nursing, University of Montreal, Montreal, Canada; 5 SHERPA University Institute, Montreal, Canada; 6 Division of Epidemiology, Dalla Lana School of Public Health, Faculty of Medicine, University of Toronto, Toronto, Canada; 7 Department of Health Behavior, Gillings School of Global Public Health, University of North Carolina at Chapel Hill, Chapel Hill, USA; 8 Department of Social and Behavioral Sciences, Harvard T.H. Chan School of Public Health, Harvard University, Boston, USA

**Keywords:** Immigrants, Discrimination, Race, Immigrant health, Health inequities, Racism, Canada

## Abstract

We examined the link between discrimination and self-rated mental health (SRMH) among immigrants and Canadian-born individuals, stratified according to an individual’s identification as racialized or white. Using data from Canada’s General Social Survey (2014) (weighted *N* = 27,575,000) with a novel oversample of immigrants, we estimated the association of perceived discrimination with SRMH separately among immigrants and Canadian-born individuals and stratified by racialized status. Among immigrants, we also investigated whether age-at-arrival attenuated or strengthened associations. The prevalence of discrimination was higher among racialized compared to white immigrants (18.9% versus 11.8%), and among racialized compared to white non-immigrants (20.0% versus 10.5%). In the adjusted model with immigrants, where white immigrants not reporting discrimination were the referent group, both white (adjusted prevalence odds ratio [aPOR] 6.11, 95% confidence interval [CI] 3.08, 12.12) and racialized immigrants (aPOR 2.28, 95% CI 1.29, 4.04) who experienced discrimination reported poorer SRMH. The associations were weaker among immigrants who immigrated in adulthood. In the adjusted model with non-immigrants, compared to unexposed white respondents, Canadian-born white respondents who experienced discrimination reported poorer SRMH (aPOR 3.62, 95% CI 2.99, 4.40) while no statistically significant association was detected among racialized respondents (aPOR 2.24, 95% CI 0.90, 5.58). Racialized respondents experienced significant levels of discrimination compared to white respondents irrespective of immigrant status. Discrimination was associated with poor SRMH among all immigrants, with some evidence of a stronger association for white immigrants and immigrants who migrated at a younger age. For Canadian-born individuals, discrimination was associated with poor SRMH among white respondents only.

**Supplementary Information:**

The online version contains supplementary material available at 10.1007/s40615-024-02128-4.

## Introduction

Migration involves a range of experiences and circumstances, from which different health problems may arise [1]. One important condition is discrimination, defined as negative attitudes, judgement or treatment of people by institutions or individuals [2], which can take several different forms according to an individual’s positioning within systems of inequality [3]. Immigrants may experience discrimination based on multiple factors, including race or skin colour, immigration status, country of origin, gender expression, sexual orientation, income, language, religion, and/or other aspects of identity [4]. There is substantial evidence linking discrimination to poor health [5, 6]. Forms of discrimination most often examined are those related to race and ethnicity, which studies have shown to be frequently associated with negative physical and mental health among immigrants [7–9]. Although Canada is a major immigrant receiving country, with migrants comprising almost a quarter of the population [10], there are sparse population-based studies that have examined links between discrimination, race/ethnicity, and immigrant health.

Canada has the second highest proportion of immigrants in the world (23%), following Australia (27%) and is one of the most important receiving countries globally [10]. Prior to 1967, the vast majority of immigrants coming to Canada were from Europe. Canada’s immigration policy during this period formally discriminated against non-white immigrants and explicitly excluded immigrants on the basis of race [11]. In the 1970s, there was a policy shift and immigration primarily became a means for the government to meet its social, humanitarian, and economic goals and with this came a move towards selecting immigrants primarily based on education levels [11]. Over time, this has resulted in significant changes in terms of source countries, with the majority of migrants today originating from Asia, Africa, the Caribbean, and South America; many of these migrants are classified as “visible minorities” by the Canadian government [11, 12]. By international standards, attitudes of the non-immigrant population in Canada towards immigrants have been relatively supportive compared to European countries and the US, with surveys consistently showing an openness to accommodate minority groups; however, experiences of discrimination and racism have also emerged as important issues [11–15].

A well-established body of literature has documented the relationship between perceived discrimination and health among immigrants, with findings generally showing a range of deleterious effects on health [7–9]. Experiences of discrimination can be stress-inducing events through overlapping direct and indirect pathways [16, 17]. For example, discrimination may directly affect psychological distress and activate physiological stress responses, leading to weakened metabolic and immune systems over time, thereby making people more at risk of disease or infection [16]. Likewise, discrimination-related psychological distress may directly act on self-esteem and mental health, leading to unhealthy coping behaviors, which in turn, can also harm health [6, 18]. Indirectly, discrimination may also shape structural/institutional interactions and the distribution of resources, leading to hardships for immigrants and negative effects on the social determinants of health, including delays and barriers to health care, precarious employment, limited educational opportunities, and denial of adequate housing [5, 19].

The literature on discrimination and immigrant health, however, also present some complexities. For instance, some studies from the US suggest that immigrants may report fewer discriminatory events [20] and experience fewer damaging effects to their health compared with US-born counterparts of the same race or ethnicity [21–23]. Other literature has found that strength of associations between discrimination and poor health increases with longer residence in receiving countries; the hypothesis being that immigrants become more aware of differential and unfair treatment over time [5, 6, 18, 24]. Scholars have also pointed to methodological inconsistencies in this literature related to sample construction and the range of different measures used, which may contribute to diverse findings [19]. Overall, researchers have called for a more intersectional approach, including accounting for simultaneous impacts of immigration and racialization processes on newcomers, as well as closer examination of the roles of structural racism and socio-historical contexts in immigrants’ countries of origin [4, 25].

Mixed findings on discrimination experiences among immigrants are also evident in the Canadian context. For example, three population-based studies using measures of racial discrimination [26] and everyday discrimination (EDS) [13, 14] found that immigrants reported less discrimination than non-immigrants, and, further, that more established immigrants and those who experienced early socialization in Canada reported similar levels of discrimination to non-immigrants [14]. In contrast, another large-scale study examining perceived workplace discrimination found the opposite and reported higher rates of discrimination among immigrants compared with non-immigrants [27]. In addition, findings from a population-based study showed that racialized (i.e., “visible minorities” defined by the Canadian government as people who are non-Indigenous and non-white) immigrants reported high levels of discrimination regardless of length of residence in Canada, whereas among white immigrants reports of discrimination were found to decrease with longer residency [28].

The majority of research to date that has examined the relationship between discrimination and health in Canada has focused on access to health care for immigrants [29–31] and only a handful of studies have specifically examined perceived discrimination as a determinant of health among immigrant populations. One small-scale study conducted with Asian immigrants in Toronto showed a positive association between racial discrimination and depressive symptoms [32]. In another study that used population-based data, De Maio and Kemp (2010) found that perceived discrimination (based on ethnicity, culture, race or skin colour, language, or religion) among “visible minority” immigrants had a stronger association with poorer self-rated health than the association observed among white immigrants [33]. Similarly, Nakhaie and Wijesingha (2015), using 2004 population data, assessed associations between perceived discrimination related to different aspects of social identity (including ethnicity/culture, race or skin colour, language, religion, sex, sexual orientation, age, and disability) and self-rated health among racialized and white immigrant women and men [34]. They found that immigrants reported more discrimination than non-immigrants, and among immigrants, women were more likely to report discrimination than men. They also found that adverse effects of discrimination on health were greater for immigrant women than men, and among immigrant women, greater for white compared to racialized individuals [34].

In view of the reported differences in self-reported discrimination between immigrants and Canadian-born individuals [13, 14, 26] and high rates of racism-based discrimination in Canada [13] documented in previous research, identification of how differences in discrimination experiences are linked to race/ethnicity, age-at-arrival, and health could highlight areas of discrimination-related health inequities that would benefit from intervention. Our analysis marks a first step in addressing these knowledge gaps by examining the relationship between discrimination and mental health status according to racialized status in a large nationally representative sample of immigrant and Canadian-born individuals. We used nationally representative Canadian survey data from the 2014 General Social Survey (GSS), the most recent GSS survey cycle to include an oversample of immigrants. Our primary analysis estimated the association of any discrimination with self-rated mental health (SRMH) separately among immigrants and Canadian-born respondents and stratified results according to racialized status (i.e., racialized vs. white). Since it has been argued that early socialization in receiving-countries may impact immigrants’ perceptions of discrimination [14], we also assessed to what extent age-at-arrival, as a proxy for early socialization, may attenuate or strengthen these relationships among immigrants. In a secondary analysis, we aimed to distinguish between perceptions of discrimination and the perceived reasons for the discrimination [35] and estimated associations between discrimination for reasons of immigration-related aspects of social identity (vs. other aspects of identity) with SRMH by immigration and racialized statuses.

## Materials and Methods

### Data Source and Sample

We used data collected in 2014 by the Canadian General Social Survey (GSS) (Cycle 28) on Canadians’ experiences of victimization. The GSS is a voluntary household survey that is conducted by Statistics Canada every 5 years. The sampling strategy consists of complex, multi-stage sampling design aimed at obtaining representative coverage of Canadian households with a telephone number. All households with telephone numbers were ascertained through a list of registered phone numbers and a registry of all dwellings in the 10 provinces. Once a household was selected and contacted by phone, an individual 15 years or older was randomly selected to complete the survey. An oversample of immigrants and youth was added to the 2014 GSS for a more detailed analysis of these groups. Survey responses were obtained by computer-assisted telephone interviews conducted in either official language (English or French). Households without telephones (approximately 1% of the target population) were not captured. The response rate was 53% and the total sample size was 33,127 respondents. Listwise deletion was used to drop < 6.2% of respondents who had missing data on key variables of interest, including respondents who did not answer questions about experiences of discrimination and self-rated mental health, as well as those for whom immigrant or racialized status could not be determined. Indigenous respondents were not included in the analytic sample because discrimination-related stressors due to colonization are distinctive characteristics of their experiences of marginality, which differs from those of other non-immigrant and immigrant groups in Canada. The final weighted analytic sample consisted of 27,575,000 respondents (unweighted *N* = 31,080).

### Measures

The survey included questions about the nature and extent of respondents’ victimization, including experiences of discrimination, as well as questions about socio-demographic characteristics, and mental and physical health. Based on prior research [35], our main exposures of interest were constructed to distinguish between the overall experience of discrimination and perceived reasons for the discrimination.

#### Exposures of Interest

Any Perceived Discrimination in the Past 5 Years: Any perceived discrimination was assessed based on a series of GSS questions about any perceived discrimination or unfair treatment for the following reasons: (1) culture/ethnicity; (2) language; (3) race/skin colour; (4) religion; (5) physical appearance; (6) biological sex; (7) sexual orientation; (8) age; (9) physical or mental disability; or (10) other reason(s). “Any perceived discrimination” was constructed as a summary variable; an affirmative response to any of the questions was categorized as “yes” (versus no discrimination). This global measure of discrimination used in the GSS is considered a primary source for information on self-perceived discrimination in Canada [36]; however, it is not widely used in the health literature, which has predominantly used original and modified versions of the Everyday Discrimination Scale (EDS) [35].

Perceived Reasons for Discrimination: Reasons for discrimination were assessed based on respondents’ answers to 10 questions about whether in the past 5 years they attributed discrimination or unfair treatment to specific reasons. Due to cell size constraints, we could not complete analyses for each reason separately and so derived two summary binary variables. The first variable combined a selection of listed reasons that we thought were likely to be more specific to the contexts of immigrants. Respondents who answered “yes” to any discrimination for reasons of culture/ethnicity, language, race/skin colour, and/or religion were considered to have experienced “immigrant-specific” discrimination (versus “no” to these reasons or no discrimination experienced in the last 5 years). The second variable combined reasons for discrimination that we thought were not specific to the experience of immigration but broadly applicable to all. In this variable, respondents who answered “yes” to discrimination for reasons of physical appearance, biological sex, sexual orientation, age, physical or mental disability, and/or other reason(s) were considered to have experienced “discrimination for other reasons” (versus “no” to these reasons or no discrimination experienced in the last 5 years).

#### Outcome

Self-rated Mental Health (SRMH): The outcome of interest was self-rated mental health measured by the single survey item: “in general, how would you rate your mental health?” with answer options of “excellent”, “very good”, “good”, “fair”, or “poor”?’ Although constructed as a five-point scale, this item is frequently dichotomized as “excellent, very good, or good health” versus “fair or poor” and in our analysis was dichotomized following this approach. As a measure, SRMH has been shown to be a reliable population health measure that is associated with multi-item measures of mental health that are not necessarily specific to a single health disorder [37]. This global assessment of mental health status applicable to various racial/ethnic groups has been posited as an especially important indicator of mental health for immigrants because it does not rely on symptoms according to Western conceptualizations of mental health disorders [38].

#### Covariates

Immigrant Status: Immigrant status (yes/no) was assessed based on respondents’ report of being born outside of Canada and obtaining legal permanent residency/citizenship. Respondents who did not report permanent residency, but who reported being born outside of Canada and provided a year for when they first came to live in Canada, were also considered immigrants, since they were likely to have arrived in Canada as asylum seekers, students or temporary workers.

Racialized status: Racialized status was constructed as a dichotomous variable. Respondents were asked (yes/no) if they identified as a “visible minority”. Respondents who self-identified as “visible minorities” were classified as “yes” (racialized) and respondents who identified as “not a visible minority” were considered “non-racialized” and are referred to as “white” throughout the manuscript. Visible minorities are defined by the Government of Canada as “persons, other than Aboriginal peoples, who are non-Caucasian in race or non-white in colour” [39]. Due to sample size constraints, we were not able to further stratify by specific racial or mixed racial groups.

Other Covariates: Several socio-demographic characteristics included in the full models were treated as potential confounders based on a priori justification of their associations with the primary exposure of perceived discrimination and the outcome of SRMH among the unexposed. Each model was adjusted for age (5-year categories), biological sex (male vs. female), educational attainment (high school completion vs. less than high school), marital status (categories: married/common law; widowed/separated/divorced; single/never married), and household income (categories: unknown, < $20,000; $20,000–39,999; $40,000 to $59,999; $60,000–$79,999, $80,000–$99,999; ≥ $100,000). For immigrant models, we additionally adjusted for length of residency (< 5 years, 5–9 years, 10 + years). For descriptive purposes only, we also included variables assessing racial/ethnic identities of respondents and whether a respondent reported a mental health condition.

### Statistical Analyses

We compared the distribution of socio-demographic characteristics and prevalence of discrimination by immigration and racialized statuses. To estimate whether and to what extent the association of perceived discrimination and SRMH was modified by racialized status, we used logistic regression models including a product term discriminated*racialized. For immigrants, we separately fit six models, one crude and one adjusted model for each discrimination-related exposure (any perceived discrimination; discrimination for reasons related to culture/ethnicity, race/skin colour, language or religion; discrimination for other reasons). In each model, we estimated stratum-specific crude and adjusted prevalence odds ratios for unexposed racialized immigrant, exposed white immigrants and exposed racialized immigrant compared with unexposed white immigrants. Because of our stratification by racialized status and prior research showing that racialized individuals in Canada reported a greater number of discrimination experiences [13], we also included a product term for discriminated*racialized. For Canadian-born individuals, we fit the same six models. Since less than 1.3% of respondents in our sample had missing data, we performed a complete case analysis in models to handle missing values in adjustment variables. To assess influences of the timing of immigrants’ exposure to the racialized structure of Canada, we fit two additional adjusted models among immigrants to assess whether discrimination was more strongly associated with fair/poor SRMH among immigrants who experienced early socialization in Canada. This additional analysis was conducted in sub-samples of immigrants who immigrated as children/youth (< 25 years old) versus adulthood (25 + years old) and also included a product term for any discrimination*racialized status. Based on previous research suggesting that early socialization in receiving-countries may modify immigrants’ perceptions of discrimination [14], we also tested a three-way product term any discrimination*racialized status*age-at-arrival. All regression models applied bootstrapped sampling weights to account for complexity of the survey design and to produce nationally representative estimates. To avoid over-adjustment of multivariable models, multicollinearity was assessed using variance inflation factors.

In a supplementary analysis, we estimated crude and adjusted prevalence odds ratios of the association between discrimination in different contexts ( e.g., in a store, bank or restaurant; at work or when applying for a job or promotion; when dealing with authorities -police, courts, border officials) and SRMH by racialized and immigration statuses.

Data were analyzed from October 8, 2019 to September 1, 2023. All data management and analyses were performed using SAS® version 9.4. The study obtained ethics approval from the Research Ethics Board at the University of Manitoba on January 9, 2019 (Protocol reference: H2018:438 (HS22337).

## Results

In the overall weighted study population (*N* = 27,575,000), white immigrant and non-immigrant (i.e., Canadian-born) individuals were more likely to be in the highest income bracket and of older age compared to racialized respondents (Table 1). The proportion of those with a high school education or higher was greatest for racialized immigrants, followed by white immigrants, white non-immigrants, and racialized non-immigrants. Length of residence in Canada was almost double for white immigrants (mean 34.8 years) compared to racialized immigrants (mean 18.0 years). The frequency of mental health conditions was lowest among racialized immigrants (7.3%) and highest among white non-immigrant respondents (14.5%), with similar proportions observed among white immigrants (11.0%) and racialized non-immigrant respondents (12.0%).

**Table 1:** Weighted sample characteristics stratified by immigrant status and racialized status, general social survey, Government of Canada, 2014

	Immigrants		Non-Immigrants	
	White	Racialized	White	Racialized
	*n* = 2,734,000	*n* = 3,766,000	*n* = 19,988,000	*n* = 1,087,000
	% of *n*	% of *n*	% of *n*	% of *n*
Main exposure				
Any experience of discrimination	11.8	18.9	10.5	20.0
Reasons for discrimination				
Reason for discrimination is due to race or skin colour, culture ethnicity, religion or language	7.8	16.7	4.9	16.7
Reason for discrimination is skin colour/race	2.3	11.6	2.0	15.3
Reason for discrimination is culture/ethnicity	4.6	11.7	2.0	14.4
Reason for discrimination is religion	2.1	4.3	1.2	4.2
Reason for discrimination is language	2.9	6.3	1.8	1.8
Reason for discrimination is due to physical appearance, sexual orientation, age, sex, disability or other	6.7	9.3	7.6	10.9
Circumstances of discrimination				
In a store, bank or restaurant	3.3	9.9	3.9	9.7
At work or when applying for a job or promotion	6.6	10.8	5.4	7.7
When dealing with authorities (police, court, or border)	1.8	3.9	0.9	6.1
Other circumstances	3.4	4.1	3.1	7.9
Socio-demographic characteristics				
Racial/ethnic identities				
Arab/White	1.1	8.0	0.1	5.8
Black		13.3		19.2
Chinese		19.5		25.3
Filipino		13.4		5
Japanese		0.8		3.9
Korean		1.9		1.0
Latin American/White	1.2	8.8	0.05	8.6
South Asian		23.3		20.1
Southeast Asian		7.4		9.9
West Asian/White	0.3	4.0		1.8
White	97.4	1.1	100.0	12.6
Other		1.0		3.8
Sex				
Male	49.0	49.4	49.2	50.9
Female	51.0	50.6	50.8	49.1
Age				
15–24	5.2	12.8	15.0	48.8
25–34	10.5	23.3	16.0	28.4
35–44	14.7	22.5	15.1	11.9
45–54	20.5	20.8	17.6	5.3
55–64	16.0	12.6	17.3	3.4
65 +	33.1	8.0	19.0	2.2
Age at arrival				
Under 25 years old	35.9	47.5		
25 years old or older	62.9	50.8		
Missing	1.2	1.7		
Length of residence (immigrants only)				
mean (SD)	34.8 (593.9)	18.0 (390.5)		
median (IQR)	37 (16–51)	15 (8–25)		
< 5 years	4.0	11.3		
5–9 years	8.0	18.2		
10 + years	86.8	69.1		
Missing	1.2	1.6		
Household income				
Under $20,000	2.9	3.0	3.1	1.5
$20,000 to $39,999	10.8	7.3	8.6	2.9
$40,000 to $59,999	11.8	8.2	10.4	4.8
$60,000 to $79,999	9.8	7.6	10.4	4.7
$80,000 to $99,999	8.3	6.9	9.5	3.6
$100,000 or more	27.5	18.7	30.8	24.8
Unknown	28.9	48.3	27.2	57.7
Educational attainment				
Less than high school	11.6	7.7	15.6	20.1
More than high school	87.5	91.5	83.8	79.6
Missing	0.9	0.8	0.6	0.3
Marital status				
Married	64.9	62.6	64.9	62.6
Common law	6.8	3.5	6.8	3.5
Widowed, separated or divorced	15.1	6.2	15.1	6.2
Single, never married	13.1	27.7	13.1	27.7
Missing	0.1	0.02	0.1	0.02
Mental health condition				
Yes	11.0	7.3	14.5	12.0
No	88.6	92.5	85.2	87.8
Missing	0.4	0.2	0.3	0.1

### Prevalence of Any Perceived Discrimination, Reasons for Perceived Discrimination, and Circumstances of Perceived Discrimination

Perceived discrimination was almost twice as high among racialized immigrant (18.9%) and non-immigrant (20.0%) respondents compared with white immigrant (11.8%) and white non-immigrant (10.5%) respondents (Table 1). Racialized immigrants (16.7%) were two times as likely to report discrimination for “immigrant-specific” reasons including race/skin colour, culture/ethnicity, religion, and/or language, compared with white immigrants (7.8%). Among specific reasons for discrimination, racialized immigrants were more than five times as likely to report discrimination for reasons related to race/skin colour (11.6 vs. 2.3%) and more than two times as likely to report discrimination for reasons of culture/ethnicity compared to white immigrants (11.7 vs. 4.6%). In terms of circumstances of discrimination, compared to white immigrants, racialized immigrants were three times as likely to report routine day-to-day discrimination (e.g., in store, bank or restaurant) (9.9 vs. 3.3%), more than two times as likely to report discrimination by authorities (3.9 vs. 1.8%), and almost two times as likely to experience discrimination in the workplace (10.8 vs. 6.6%).

### Discrimination, Reasons for Discrimination, and Mental Health Among Non-immigrant Individuals

White non-immigrant respondents who experienced discrimination reported a greater odds of poor SRMH (aPOR 3.62, 95% CI 2.99, 4.40) than non-immigrant racialized respondents (aPOR 2.24, 95% CI 0.90, 5.58), the latter for whom no statistically significant association was detected (Table 2; reference group is white non-immigrant individuals unexposed to discrimination). A similar pattern held among non-immigrant respondents who attributed their discrimination to reasons of culture/ethnicity, race/skin colour, language and/or religion, where worse SRMH was reported among non-immigrant white respondents (aOR 2.51, 95% CI 1.91, 13.30), and no statistically significant association was observed among racialized respondents (aOR 1.88, 95% CI 0.39, 9.70) (Table 3; reference group is white Canadian-born individuals unexposed to these types of discrimination or having no discrimination experience). The pattern differed however among non-immigrant respondents who attributed their discrimination as being due to other reasons (i.e., physical appearance, biological sex, sexual orientation, age, physical or mental disability, and/or other), where our results showed that both white (aPOR 3.93, 95% CI 3.19, 4.84) and racialized (aPOR 4.20, 95% CI 1.57, 11.21) non-immigrant respondents had a similarly high likelihood of poor SRMH.

**Table 2:** Crude and adjusted weighted odds ratios of association of any discrimination and poor/fair self-rated mental health by immigrant status and age at migration, General Social Survey, Government of Canada, 2014

	**Non-Immigrants**					**All immigrants**					**Immigrated < age 25**			**Immigrated ≥ age 25**		
	% Poor/fair mental health	POR	95% CI	aPOR*	95% CI	% Poor/fair mental health	POR	95% CI	aPOR**	95% CI	% Poor/fair mental health	aPOR*	95% CI	% Poor/fair mental health	aPOR*	95% CI
Any discrimination																
No																
White (ref.)	4.3	1.00	ref	1.00	ref	3.4	1.00	ref	1.00	ref	3.3	1.00	ref	3.5	1.00	ref
Racialized	4.7	1.10	0.61, 1.97	0.87	0.48, 1.57	2.3	0.67	0.44, 1.02	0.75	0.47, 1.22	3.0	1.01	0.54, 1.90	1.5	0.51	0.22, 1.18
Yes																
White	17.8	3.87	3.22, 4.66	3.62	2.99, 4.40	17.0	5.81	2.99, 11.28	6.11	3.08, 12.12	20.5	8.30	3.62, 19.04	9.1	3.15	1.03, 9.64
Racialized	11.1	2.79	1.12, 6.94	2.24	0.90, 5.58	6.8	2.08	1.31, 3.29	2.28	1.29, 4.04	7.9	2.57	1.22, 5.39	5.5	2.12	0.78, 5.77
* p*-value for interaction		0.4		0.5			0.1		0.1			0.02			0.7	
(any discrimination*racialized status)																

*Model is adjusted age, sex, educational attainment, household income, marital status

**Model is adjusted age, sex, educational attainment, household income, marital status, length of residence

*POR* prevalence odds ratio, *aPOR* adjusted prevalence odds ratio, *CI* confidence interval, *ref*. reference.

**Table 3:** Crude and adjusted weighted odds ratios of association of reasons for discrimination and poor/fair self-rated mental health by immigrant status. General Social Survey, Government of Canada, 2014

	**Non-Immigrants**					**All immigrants**				
	% Poor/fair mental health	POR	95% CI	aPOR**	95% CI	% Poor/fair mental health	POR	95% CI	aPOR*	95% CI
Discrimination due to race/skin, culture/ethnicity, language or religion										
No										
White (ref.)	5.1	1.00	ref	1.00	ref	4.2	1.00	ref	1.00	ref
Racialized	4.9	0.97	0.56, 1.65	0.73	0.43, 1.26	2.4	0.55	0.37, 0.81	0.60	0.38, 0.94
Yes										
White	11.9	2.55	1.96, 3.31	2.51	1.91, 3.30	14.1	3.74	1.25, 11.23	4.17	1.32, 13.16
Racialized	11.5	2.44	0.51, 11.72	1.88	0.39,9.07	7.0	1.72	1.08, 2.73	1.80	1.02, 3.19
* p*-value for interaction		0.99		0.99			0.8		0.6	
Discrimination due to other factors^+^										
No										
White (ref.)	7.8	1.00	ref	1.00	ref	3.7	1.00	ref	1.00	ref
Racialized	6.5	0.98	0.56, 1.73	0.77	0.43, 1.37	2.60	0.71	0.48, 1.05	0.77	0.48, 1.23
Yes										
White	16.8	4.30	3.54, 5.24	3.93	3.19, 4.84	23.1	7.89	3.53, 17.61	7.77	3.45, 17.53
Racialized	19.7	5.25	1.97, 14.02	4.20	1.57, 11.21	8.0	2.26	1.37, 3.76	2.27	1.24, 4.14
* p*-value for interaction		0.7		0.6			0.06		0.05	

*Model is adjusted age, sex, educational attainment, household income, marital status

**Model is adjusted age, sex, educational attainment, household income, marital status, age at arrival

+Other factors include discrimination is due to physical appearance, sexual orientation, age, sex, disability or other

*POR* prevalence odds ratio, *aPOR* adjusted prevalence odds ratio, *CI* confidence interval, *ref* reference

### Discrimination, Reasons for Discrimination, and Mental Health Among Immigrants

Among immigrants, white immigrants who experienced discrimination reported the highest odds of worse SRMH (aPOR 6.11, 95% CI 3.08, 12.12) followed by racialized respondents who experienced discrimination (aPOR 2.28, 95% CI 1.29, 4.04) (Table 2; reference group is white immigrants unexposed to discrimination). Further, while a secondary analysis (not shown) did not find strong statistical evidence of a three-way interaction with discrimination, racialized status, and age-at-arrival (interaction *p*-value 0.10), a decision to stratify results by age-at-arrival was made based on theoretical knowledge of early socialization. In stratified results, strong evidence of modification by racialized status (interaction *p*-value: 0.02) was observed in the subsample of immigrants who were 25 years or younger upon arrival to Canada. Among white immigrants, respondents who immigrated at younger ages and experienced discrimination had a prevalence of poor SRMH that was more than twice the prevalence of their counterparts who immigrated above age 25 years (20.5 vs. 9.1%); whereas, a far smaller difference in prevalence was observed between racialized respondents who were 25 years or younger at immigration compared to above age 25 years (7.9 vs. 5.5%). In the adjusted model for immigrants who arrived at 25 years or younger (Table 2; reference group is white immigrants unexposed to discrimination), white immigrants who experienced discrimination had the highest odds of poor SRMH (aPOR 8.30, 95% CI 3.62, 19.04) followed by racialized immigrants who experienced discrimination (aPOR 2.57, 95% CI 1.22, 5.39). Among immigrants who arrived above age 25, white immigrants who experienced discrimination had a high odd of poor SRMH (aPOR 3.15, 95% CI 1.03, 9.64), while there was no statistically significant association detected among racialized immigrants (aPOR 2.12, 95% CI 0.78, 5.77).

 White immigrants who experienced discrimination due to “immigrant-specific” reasons had the highest odds of worse SRMH (aOR 4.17; 95% CI 1.32, 13.16) followed by exposed racialized respondents (aOR 1.80; 95% CI 1.02, 3.19), and unexposed racialized respondents (aPOR 0.60, 95% CI 0.38, 0.94) (Table 3; reference group is white immigrants unexposed to these types of discrimination or experiencing no discrimination). There was evidence of the same graded relationships among immigrants who were discriminated against for other reasons (i.e., physical appearance, biological sex, sexual orientation, age, physical or mental disability, and/or other), where white immigrants had the highest odds of worse SRMH (aPOR 7.77, 95% CI 3.45, 17.53), followed by exposed racialized immigrants (aPOR 2.27, 95% CI 1.24, 4.14).

In the supplementary analysis of circumstance-specific discrimination (Supplementary Table 1), results showed some evidence of worse SRMH for white immigrants in each setting (i) in a store, bank or restaurant; (ii) at work or when applying for a job or promotion; (iii) when dealing with authorities -police, courts, border officials compared with racialized immigrants. In the Canadian-born sample, racialized and white respondents had similarly high odds of poor SRMH, with the exception of discrimination in a store, bank, or restaurant, where results showed no statistically significant association with SRMH detected among racialized respondents***.***

## Discussion

This study examined the association between experiences of perceived discrimination and self-rated mental health stratified by racialized status in a large nationally representative sample of immigrant and Canadian-born individuals in Canada. Overall, we found that racialized respondents regardless of immigrant status experienced vastly higher levels of discrimination across all measures, particularly when discrimination was perceived as due to race/skin colour and culture/ethnicity, and that the largest difference in rates of discrimination based on racialized status was observed among Canadian-born individuals. We also found that while discrimination was associated with poor SRMH, there was some evidence that this association was weaker among racialized respondents who experienced the highest levels of discrimination. For example, among Canadian-born individuals, the odds of poor SRMH were relatively high for white respondents who experienced any discrimination; whereas among racialized respondents, the association between discrimination and SRMH was weaker and limited to discrimination for reasons of physical appearance, sexual orientation, age, sex, disability, or other. In contrast, among immigrants, the association between discrimination and poor SRMH was consistent across discrimination measures for all immigrants, but was generally higher among white immigrants. In further results stratified by age-at-arrival, the association between discrimination and poor mental health was significant among white and racialized immigrants who immigrated at a younger age and weaker among counterparts who arrived at an older age.

The findings of significantly high levels of discrimination based on race/skin colour and culture/ethnicity in our study expand on previous research confirming the frequency of discrimination faced by both racialized and immigrant peoples in Canada [14, 26], and also bring into focus the embedded structures and practices of white supremacy that contribute to these experiences [40, 41]. For racialized immigrants in particular, our findings showing high levels of discrimination are consistent with patterns of rising xenophobia and racialized immigration enforcement in Canada [42]. Our additional finding of a narrower gap in rates of perceived discrimination between white and racialized immigrants compared with Canadian-born individuals is also notable and may be partly attributable to the inadequacy of discrimination indicators capturing the discrimination experiences of racialized immigrants. Future research study designs in which individual-level perceptions of discrimination are included in tandem with measures of structural and institutional policies that can foster racial discrimination (e.g., housing, education, employment) could offer promising new insights in this area [4, 25].

In agreement with prior research from Canada [13, 34], our results show that the association between discrimination and poor health was more pronounced for white immigrants compared with racialized immigrants. There are several potential overlapping explanations for these findings. One explanation could be the study outcome measure of SRMH, which may be misclassifying mental health status based on racial/ethnic and cultural differences in conceptualizations of mental health and norms related to the reporting of mental health problems. A second reason relates to our sample size, which did not allow for direct analysis of the association between racial discrimination and mental health, thus potentially underestimating the true effects of race-based discrimination on the health of racialized respondents. A third explanation may also be related to our collapsing of different racialized groups into one category, whereby there is likely dilution of the health effects of discrimination for some racialized individuals, such as Black individuals, for whom experiences of discrimination include the manifold systemic effects of anti-Black racism. A fourth explanation, which should be considered cautiously to not distract from the enormous harms of racism, could also lie within white supremacist ideologies. In this supremacist belief system where “whiteness” is centered as dominant in the social hierarchy, white respondents may be more likely to associate any experience of discrimination as a threat to “white” status, which prior research suggests can introduce a risk to their health, while also potentially leading white people to embrace supremacist ideologies that are harmful to racialized peoples [43]. To more clearly understand these relationships, future research initiatives in Canada should include investment in population-based data that enable robust analysis of the effects of racism on health among both immigrant and Canadian-born populations, including exploration of differences in measures of mental disorders in different racial and ethnic groups. This field of research is especially salient in light of the rise of white supremacist violence in Canada. This violence, observed in increasing anti-Muslim hate incidents, notably the Quebec City mosque massacre (2017) and targeted car attack and killing of Muslim pedestrians (2021), the rise in anti-Asian hate incidents during the pandemic (2020), and the growing roots of transnational far-right movements in Canada displayed in the recent Freedom Convoy protests in Ottawa (2022) [44, 45], also creates a more enabling environment for harmful and restrictive immigration policies that future research agendas focused on immigrant health should prioritize [25].

Our results showing stronger associations among white and racialized immigrants who experienced discrimination and arrived in Canada before age 25 years suggest some evidence in support of the influence of early socialization [14]; but other explanations are also possible. For example, research has suggested that under-reporting of discrimination by immigrants who arrived in adulthood is common for a variety of reasons, including social desirability [5, 46, 47]. It is also plausible that global discrimination indicators are inadequate in capturing the potentially subtle and recurrent experiences of discrimination among individuals who immigrated later in life [48]. Future work is warranted to better understand how immigrants perceive and react to discrimination in different contexts, such as attention to immigrants’ cumulative experiences of discrimination over time, including discrimination in their countries of origin.

Our study has several limitations. First, due to sparse data we were not able to parse out the discrimination–SRMH relationship in specific racial groups. Since the majority of racialized respondents in our study identified as being Chinese, Filipino, and South Asian, our analysis is thus likely under-estimating the health effects of discrimination on Black individuals whose experiences encompass the systemic effects of anti-Black racism, and potentially biasing results toward the null. Second, we used cross-sectional data, which implies the temporal ordering and directions of the associations are unknown. A longitudinal study design that can assess these relationships would be helpful for refining directionality and causal processes. Third, while we assessed discrimination globally, as well as reasons for discrimination groups according to immigration-related aspects of social identity (vs. other reasons), and several different circumstances of discrimination, our sample size constraints precluded examination of the direct association of racial discrimination and mental health, thus potentially underestimating the true association of discrimination and mental health among racialized respondents. Fourth, based on previous evidence of differential validity and reliability of self-reported discrimination by socio-demographic characteristics [49], it is likely that perceptions of and willingness to report discrimination varies among respondents in our study. These measures may therefore introduce bias, while also not accounting for the anticipation or persistence of discrimination experiences. Qualitative and quantitative studies that incorporate assessments of structural racism and the presence or absence of discrimination-related stressors, such as vigilance, may help improve internal validity of future research in this area. Fifth, due to English/French language restrictions of the GSS, more marginalized immigrants, such as refugees, for whom forced migration has been associated with lower fluency with Canada’s official languages, are more likely to have been excluded. Finally, we recognize that Canada has a history of supportive attitudes towards immigrants relative to other jurisdictions, and in this context, the external validity of our study may be limited.

## Conclusion

Our study shows that racialized respondents experienced significant levels of discrimination compared to white respondents irrespective of immigrant status. The experience of any discrimination was associated with poor mental health among all immigrants, with some evidence of a stronger association for white immigrants. Findings bolster calls for broader attention to the links between anti-immigrant sentiment, racism, and the health of immigrants, including empirical characterization of how structural racism and interpersonal racism intersect to uniquely impact the health of immigrants. Findings also support calls for the development of population-based survey data on racial discrimination in Canada to enable more robust analysis of the effects on health among both immigrant and Canadian-born populations, and inform development of interventions focused on reducing discrimination-related health inequities.

## Supplementary Information

Below is the link to the electronic supplementary material.

Supplementary file1 (DOCX 29 KB)

## Data Availability

The data that support the findings of this study are available from *Statistics Canada* but restrictions apply to the availability of these data, which were used with approvals for the current study, and so are not publicly available.

## Code Availability

Code used for this study cannot be made publicly available due to sharing restrictions by *Statistics Canada*.
