# Supplementary material for: Discrimination and Racial Inequities in Self-reported Mental Health Among Immigrants and Canadian-Born Individuals in a Large, Nationally Representative Canadian Survey
Source: J Racial Ethn Health Disparities. 2024 Aug 20;12(5):3240–52. doi: 10.1007/s40615-024-02128-4 (PMC12446120; doi:10.1007/s40615-024-02128-4)
Supplement: Supplementary file 1 — Supplementary file1 (DOCX 29 KB) [file 40615_2024_2128_MOESM1_ESM.docx]

| **Supplementary Table 1:** Crude and adjusted weighted odds ratios of association of discrimination and poor/fair self-rated mental health by immigrant status, General Social Survey, Government of Canada, 2014. | | | | | | | | | | | | |
| --- | --- | --- | --- | --- | --- | --- | --- | --- | --- | --- | --- | --- |
|  |  | **Non-Immigrants** | | | | |  | **Immigrants** | | | | |
|  | | Prevalence poor/fair mental health | POR | 95% CI | aPOR* | 95% CI |  | Prevalence poor/fair mental health | POR | 95% CI | aPOR** | 95% CI |
| **Discrimination in a store, bank or restaurant** | | |  |  |  |  |  |  |  |  |  |  |
| No | |  |  |  |  |  |  |  |  |  |  |  |
| White (ref.) | | 5.1 | 1.00 | ref. | 1.00 | ref. |  | 4.7 | 1.00 | ref. | 1.00 | ref. |
| Racialized | | 4.6 | 0.91 | 0.54, 1.54 | 0.69 | 0.40, 1.18 |  | 2.7 | 0.57 | 0.39, 0.84 | 0.59 | 0.38, 0.92 |
| Yes | |  |  |  |  |  |  |  |  |  |  |  |
| White | | 14.0 | 3.06 | 2.31, 4.04 | 2.78 | 2.08, 3.70 |  | 12.5 | 2.88 | 1.09, 7.61 | 3.11 | 1.16, 8.33 |
| Racialized | | 18.3 | 4.21 | 0.82, 21.57 | 3.21 | 0.62, 16.49 |  | 6.6 | 1.44 | 0.79,2.62 | 1.40 | 0.71, 2.80 |
| p-value for interaction | |  | 0.6 |  | 0.6 |  |  |  | 0.8 |  | 0.7 |  |
| (any discrimination*racialized status) | | |  |  |  |  |  |  |  |  |  |  |
| **Discrimination at work or when applying for a job or promotion** | | |  |  |  |  |  |  |  |  |  |  |
| No |  |  |  |  |  |  |  |  |  |  |  |  |
| White (ref.) | | 5.0 | 1.00 | ref. | 1.00 | ref. |  | 4.1 | 1.00 | ref. | 1.00 | ref. |
| Racialized | | 4.6 | 0.92 | 0.54, 1.57 | 0.70 | 0.40, 1.20 |  | 2.6 | 0.62 | 0.41, 0.92 | 0.66 | 0.41, 1.08 |
| Yes | |  |  |  |  |  |  |  |  |  |  |  |
| White | | 13.3 | 2.95 | 2.33, 3.74 | 2.99 | 2.33, 3.84 |  | 17.9 | 5.12 | 1.95, 13.41 | 6.11 | 2.27, 16.47 |
| Racialized | | 22.6 | 5.62 | 1.72, 18.36 | 4.66 | 1.44, 15.08 |  | 7.8 | 1.99 | 1.21, 3.27 | 2.21 | 1.23, 3.95 |
| p-value for interaction | |  | 0.3 |  | 0.2 |  |  |  | 0.4 |  | 0.3 |  |
| (any discrimination*racialized status) | | |  |  |  |  |  |  |  |  |  |  |
|  |  |  |  |  |  |  |  |  |  |  |  |  |
|  |  |  |  |  |  |  |  |  |  |  |  |  |
| **Discrimination by authorities (police, court, border)** | | |  |  |  |  |  |  |  |  |  |  |
| No |  |  |  |  |  |  |  |  |  |  |  |  |
| White (ref.) | | 5.2 | 1.00 | ref. | 1.00 | ref. |  | 4.3 | 1.00 | ref. | 1.00 | ref. |
| Racialized | | 4.6 | 0.88 | 0.53, 1.48 | 0.67 | 0.40, 1.13 |  | 2.8 | 0.64 | 0.45 0.92 | 0.67 | 0.43, 1.04 |
| Yes | |  |  |  |  |  |  |  |  |  |  |  |
| White | | 26.4 | 6.52 | 4.15, 10.25 | 5.61 | 3.50, 9.00 |  | 40.3 | 14.92 | 2.50 , 89.21 | 13.80 | 2.07, 91.86 |
| Racialized | | 29.5 | 7.60 | 1.84, 31.40 | 6.28 | 1.54, 25.64 |  | 9.9 | 2.44 | 1.26, 4.71 | 2.25 | 1.08, 4.70 |
| p-value for interaction | |  | 0.7 |  | 0.5 |  |  |  | 0.2 |  | 0.2 |  |
| (any discrimination*racialized status) | | |  |  |  |  |  |  |  |  |  |  |
| **Discrimination by other** | |  |  |  |  |  |  |  |  |  |  |  |
| No | |  |  |  |  |  |  |  |  |  |  |  |
| White (ref.) |  | 4.8 | 1.00 | ref. | 1.00 | ref. |  | 4.5 | 1.00 | ref. | 1.00 | ref. |
| Racialized |  | 5.6 | 1.17 | 0.72, 1.93 |  |  |  | 3.0 | 0.66 | 0.45, 0.98 | 0.69 | 0.43, 1.11 |
| Yes |  |  |  |  |  |  |  |  |  |  |  |  |
| White |  | 22.6 | 5.72 | 4.39, 7.46 | 4.75 | 3.59, 6.28 |  | 3.0 | 0.66 | 0.45, 0.98 | 3.38 | 1.29, 8.86 |
| Racialized |  | 9.9 | 2.15 | 0.047, 98.68 | 1.61 | 0.04, 74.21 |  | 5.3 | 1.20 | 0.50, 2.87 | 0.96 | 0.36, 2.52 |
| p-value for interaction | |  | 0.6 |  | 0.6 |  |  |  | 0.2 |  | 0.2 |  |
| (any discrimination*racialized status) | | |  |  |  |  |  |  |  |  |  |  |
|  | | |  |  |  |  |  |  |  |  |  |  |
|  | | |  |  |  |  |  |  |  |  |  |  |
|  | | | | | | | | | | | | |
|  | | | | | | | | | | | | |
|  | | |  |  |  |  |  |  |  |  |  |  |
|  | | | | | | | | | | | | |
|  | | | | | | | | | | |  |  |

*Model is adjusted age, sex, educational attainment, household income, marital status

**Model is adjusted age, sex, educational attainment, household income, marital status, age at arrival

Abbreviations: POR: prevalence odds ratio, aPOR: adjusted prevalence odds ratio, CI, confidence interval

| **Supplementary Table 2:**  Geographic sub-region of white immigrants respondents, General Social Survey, Government of Canada, 2014. | | | |
| --- | --- | --- | --- |
|  | Immigrants | | |
|  | White (unweighted) | |  |
|  | n | % |  |
| North America | 299 | 9.6 |  |
| Central America | 43 | 1.4 |  |
| Caribbean and Bermuda | 34 | 1.1 |  |
| South America | 78 | 2.5 |  |
| Western Europe | 546 | 17.5 |  |
| Eastern Europe | 562 | 18.0 |  |
| Northern Europe | 788 | 25.2 |  |
| Southern Europe | 398 | 12.7 |  |
| Western, Central, Southern Africa | 71 | 2.3 |  |
| Eastern Africa | 15 | 0.5 |  |
| Northern Africa | 52 | 1.7 |  |
| West Central Asia and Middle East | 142 | 4.5 |  |
| Eastern and Southeast Asia and Southern Asia | 29 | 1.0 |  |
| Oceania | 45 | 1.4 |  |
| Missing | 23 | 0.7 |  |
|  |  |  |  |

| **Supplementary Table 3:**  Place of birth of racialized immigrants respondents, General Social Survey, Government of Canada, 2014. | | | |
| --- | --- | --- | --- |
|  | Immigrants | |  |
|  | Racialized (unweighted) | |  |
|  | n | % |  |
| North America | 37 | 1.0 |  |
| Central America | 130 | 3.4 |  |
| Caribbean and Bermuda | 306 | 8.0 |  |
| South America | 248 | 6.5 |  |
| Western, Southern Europe | 34 | 0.9 |  |
| Eastern and Northern Europe | 38 | 1.0 |  |
| Western, Central, Southern Africa | 158 | 4.1 |  |
| Eastern Africa | 156 | 4.1 |  |
| Northern Africa | 194 | 5.0 |  |
| West Central Asia and Middle East | 368 | 9.6 |  |
| Eastern Asia | 684 | 17.8 |  |
| Southeast Asia | 758 | 19.7 |  |
| Southern Asia | 664 | 17.3 |  |
| Oceania | 33 | 0.9 |  |
| Missing | 39 | 1.0 |  |

| **Supplementary Table 4:** Age of arrival of immigrants by length of residence, unweighted, General Social Survey, Government of Canada, 2014. | | | | | | | | | | | | | |
| --- | --- | --- | --- | --- | --- | --- | --- | --- | --- | --- | --- | --- | --- |
|  | Length of residence | | | | | | | | | | | |  |
|  | Racialized immigrants | | | | | |  | White immigrants | | | | | |
|  | <5 years | | 5-9 years | | 10+ years | |  | <5 years | | 5-9 years | | 10+ years | |
|  | % | | % | | % | |  | % | | % | | % | |
| Age of arrival |  |  |  |  |  |  |  |  |  |  |  |  |  |
| Under 25 years old | 44.0 | | 57.0 | | 61.0 | |  | 29.0 | | 42.0 | | 66.0 | |
| 25 years or older | 56.0 | | 43.0 | | 39.0 | |  | 71.0 | | 58.0 | | 34.0 | |
|  |  |  |  |  |  |  |  |  |  |  |  |  |  |
